# Quercetin Alleviates Inflammation and Energy Deficiency Induced by Lipopolysaccharide in Chicken Embryos

**DOI:** 10.3390/ani13132051

**Published:** 2023-06-21

**Authors:** Jinhai Yu, Guoliang Hu, Xiaoquan Guo, Huabin Cao, Caiying Zhang

**Affiliations:** Jiangxi Provincial Key Laboratory for Animal Health, Institute of Animal Population Health, College of Animal Science and Technology, Jiangxi Agricultural University, Nanchang 330045, China; yjh6262003@126.com (J.Y.);

**Keywords:** quercetin, LPS, energy deficiency, AMPKα, APOA4, PEPT1, PPARα, SGLT1

## Abstract

**Simple Summary:**

Energy deficiency causes multiple organ dysfunctions in the lipopolysaccharide (LPS)-induced model. Quercetin is a flavonoid found in many plants including herbal medicines; nevertheless, the protective effects of quercetin in alleviating LPS-induced energy deficiency remain unclear. In the present study, an in ovo LPS-induced inflammation model was established in chicken embryos. Quercetin attenuated the increase in glycogen and lipid contents in the liver after LPS stimulation when compared with the control group. Quercetin could downregulate the mRNA expressions of AMPKα1 and AMPKα2 in the duodena, ceca, and livers when compared with the LPS group; quercetin decreased the immunoreactivity to AMPKα2 in the duodena and livers. The LPS-induced high mRNA expressions of PPARα and SGLT1 were blocked by quercetin in the duodena, ceca, and livers. Quercetin improved the APOA4 decrease in the duodena, ceca, and livers after LPS induction. Quercetin could downregulate the mRNA expression of PEPT1 in the duodena and ceca increased after LPS challenge. These data demonstrate that quercetin improves the energy deficiency induced by LPS in chicken embryos.

**Abstract:**

Energy deficiency causes multiple organ dysfunctions after LPS induction. Quercetin is a phenolic compound found in herbal medicines. However, the effects of quercetin in alleviating LPS-induced energy deficiency remain unclear. In the present study, an in vivo LPS-induced inflammation model was established in chicken embryos. Specific pathogen-free chicken embryos (n = 120) were allocated to control, PBS with or without ethanol, quercetin (10, 20, or 40 nmol, respectively), and LPS (125 ng/egg) with or without quercetin groups. Fifteen day old embryonated eggs were injected with the abovementioned solutions via the allantoic cavity. On embryonic day 19, the tissues of the embryos were collected for histopathological examination using frozen oil red O staining, RNA extraction, real-time quantitative polymerase chain reaction, and immunohistochemical investigations. The glycogen and lipid contents in the liver increased after LPS stimulation as compared with the PBS group, whereas quercetin decreased the accumulation as compared with the LPS group. The mRNA expressions of AMPKα1 and AMPKα2 in the duodena, ceca, and livers were upregulated after LPS induction as compared with the PBS group, while quercetin could downregulate these expressions as compared with the LPS group. The immunopositivity of AMPKα2 in the villus, crypt, lamina propria, tunica muscularis, and myenteric plexus in the duodena and in the cytoplasms of hepatocytes significantly increased after LPS induction when compared with the PBS group (*p* < 0.01), whereas the immunopositivity to AMPKα2 in the quercetin treatment group significantly decreased when compared with the LPS group (*p* < 0.01 or *p* < 0.05). The LPS-induced high expressions of transcription factor PPARα and glucose transporter (SGLT1) were blocked by quercetin in the duodena, ceca, and livers. Quercetin treatment improved the LPS-induced decrease in APOA4 in the duodena, ceca, and livers. The mRNA expression of PEPT1 in the duodena and ceca increased after LPS challenge, whereas quercetin could downregulate PEPT1 gene expression. These data demonstrate that quercetin improved the energy deficiency induced by LPS in chicken embryos. The LPS-induced inflammation model was established to avoid the effect of LPS exposure from the environment and intestinal flora. The results form the basis the administration of quercetin pretreatment (in ovo infection) to improve the energy state of chicken embryos and improve the inflammation response.

## 1. Introduction

The zoonotic potential of *Escherichia coli* or *Salmonella* spp. from chicken-source food products is important to define for public health purposes. *Salmonella enterica* serotype Typhimurium and *Salmonella enterica* serotype Enteritidis are the leading serovars responsible for human and animal salmonellosis globally, with the majority of human cases originating from foodborne outbreaks [1]. The common clinical manifestations of salmonellosis include diarrhea, fever, dehydration, and nausea/vomit. This indicates that the digestion and absorption of glucose, lipid, and protein in the alimentary system are affected in this condition.

In energy deficiency, cells fail to maintain electrolyte balance, membrane integrity, and protein synthesis. Adenosine monophosphate-activated protein kinase (AMPK) is a crucial intracellular energy sensor and regulator of energy homeostasis. The energy deficiency associated with sepsis presents an acute and organism-wide hyperinflammatory response and multiple organ dysfunctions [2]. One study indicated that the level of amino acids and carbohydrates decreased, while that of free fatty acids and acylcarnitines increased in the mouse sepsis model [3]. The treatment of salmonellosis constitutes antibiotics, but their overuse or abuse leads to increased drug resistance; therefore, alternative medicine is needed to deal with this problem. Active ingredients of Chinese herbal medicine may be a reasonable choice.

Quercetin, a pentahydroxyflavone, is a phenolic compound found in many plants including herbal medicines. It exhibited anti-inflammatory effects in the jejuna of broiler chicken after LPS challenge [4]. In addition, previous studies indicated that quercetin attenuates oxidative damage in embryonic chicken spermatogonial cells [5], granulosa cells from chicken ovarian follicles [6], and chicken heterophils [7]. Lipopolysaccharide (LPS), a component of the Gram-negative bacterial cell wall, has been used to induce a sepsis model in dog [8], mouse [9], and rat [10], where it induces inflammation of and energy deficiency in multiple organs, resulting in the depletion of glycogen, glucose, and ATP storage, and increases in the level of lactate, the fatty acid oxidation, and protein oxidation [11]. However, there is no report on the energy deficiency resulting from an LPS-induced model in chicken. In addition, the effects of quercetin in alleviating the LPS-induced energy deficiency in chicken remain elusive.

Intestinal epithelial cells are the first barrier function in the inflammatory response, involved in solute transport and nutrient absorption. The proton-coupled peptide transporter 1 (PEPT1) is highly expressed on the brush border of enterocytes and is involved in energy balance in mammals and chicken. PEPT1 has a high capacity and low affinity when absorbing small peptides from the small intestine in multicellular eukaryotes, and it can transport more than 8400 dipeptides or tripeptides in an acidic microenvironment (pH 4.5–6.5) [12]. There are three types of sodium–glucose transporters (SGLTs) in humans: SGLT1, SGLT2, and SGLT3. SGLT1 is expressed in the small intestine, heart, and kidney. SGLT1 has high affinity and low volume, and the transport ratio of sodium to glucose is 2:1. PEPT1 and SGLT1 mRNA is expressed between embryonic days 19 and 35 post hatch in six broiler breeds [13]. One report indicated that in ovo threonine supplementation attenuated ileal gene expression of PEPTI and SGLT1 in broilers inoculated post hatch with *Salmonella* Enteritidis [14]. Nevertheless, there is no report about the effects of in ovo quercetin injection on PEPT1 and SGLT1 mRNA expression in the duodena and ceca of the chicken embryos after LPS introduction from *Salmonella enterica* serotype Typhimurium administration.

PPAR (peroxisome proliferation-activated receptor) belongs to the steroid hormone receptor superfamily and binds to retinoid X receptors to form heterodimers that regulate fat and glucose metabolism, energy homeostasis, inflammatory response, and vascular function [15]. There are three forms of PPAR gene (PPARα, PPARβ/δ, and PPARγ), encoding PPARA, PPARD, and PPARG, respectively. PPARA is mainly found in the liver, skeletal muscle, cardiac muscle, and kidney in humans [16], regulating the ketogenesis, lipid storage, and fatty acid oxidation pathways. Evidence has shown that PPARα-knockout mice have fatty liver effects after fasting for 36 h, with elevated levels of peroxide, nitric oxide synthase, hydrogen peroxide, and protein oxidation in the liver [17]. Another study indicated that intestine-specific PPARα-null mice decreased obesity-associated metabolic disorders after feeding a high-fat diet, including lower bodyweight gain, insulin sensitivity improvement, and short length of small intestines [18]. Quercetin could downregulate the hepatic mRNA expression of PPARα in mice fed high-fat diets [19] and high-fructose rations [20]. Nevertheless, the gene expressions of PPARα in the duodena, ceca, and livers in chicken embryos after LPS stimulation with or without quercetin treatment remain elusive.

Apolipoprotein A4 (APOA4), an abundant apolipoprotein, is the main component of high-density lipoprotein and triglyceride-rich lipoprotein particles, where it plays an important role in fat transport and metabolism. APOA4 is synthesized and bound to chylomicron primarily in the small intestine, before entering into the intestinal lymph nodes, and being secreted into the intestine. When triglyceride is broken down, APOA4 converts chylomicron into high-density lipoprotein and enters into the bloodstream. Reverse cholesterol transport plays a crucial role in exporting cholesterol from peripheral cells in the prevention and treatment of atherosclerosis. It has been postulated that LPS can induce atherosclerosis [21]. One study indicated that quercetin improved macrophage reverse cholesterol transport in apolipoprotein E-deficient mice in an atherosclerosis model fed a high-fat diet [22], while another study found the quercetin could induce apolipoprotein A1 synthesis in hepatocytes [23]. There is no report on how quercetin influences APOA4 mRNA expression in the liver of chicken embryos after LPS induction.

In the present study, an LPS induction model was established to avoid the effect of LPS exposure from the environment and intestinal flora. The energy deficiency-associated gene and protein expressions were revealed after LPS induction in the duodena, ceca, and livers of the chicken embryos. The effects of quercetin on these observations and expressions in the three organs are demonstrated in this manuscript.

## 2. Materials and Methods

### 2.1. Reagents, Chicken Embryos, and Experimental Design

LPS from *Salmonella enterica* serotype Typhimurium (*S.* Typhimurium, product number: L7261, Sigma-Aldrich Trading Co., Ltd., Shanghai, China) was dissolved in phosphate-buffered solution (PBS) at 0.6 μg/mL (125 ng/egg). Quercetin (Product number: Q4591, Sigma-Aldrich Trading Co., Ltd., Shanghai, China) was dissolved in 100% ethanol at 50, 100, or 200 μM (10, 20, or 40 nmol/egg). The study was approved by the University Animal Ethics Committee (JXAULL-2022002).

Because the chick genome demonstrates remarkable evolutionary conservation with mammals, the expression patterns of several genes and proteins are well conserved between chick and mouse embryos. In addition, injection into the allantoic cavity of chicken embryos is an ideal method to avoid the exposure to environmental LPS and intestinal LPS from gut microorganisms. Therefore, chicken embryos were selected for the present study. There is ethanol dehydrogenase activity in the liver of chicken [24], and ethanol metabolism increased 20 min after intra-allantoic injection before returning to control levels [25]; hence, quercetin was dissolved in ethanol to increase its solubility. One study indicated that the mortality of chicken embryos after LPS from *Salmonella enterica* serotype Typhimurium induction with 6 mg/embryonic egg and 8 mg/embryonic egg in 19 days was 33% and 86%, respectively [26]. Nevertheless, the survival rate was 100% with a dosage of 125 ng LPS/embryonic egg in the present study. In addition, the survival rate was 100% with dosages of 500 ng and 1000 ng LPS/embryonic egg in the preliminary experiment.

Specific pathogen-free Babcock embryos (weight 56.76 ± 3.32 g) were provided by a chicken breeder (Ji’nan SAIS Poultry Co., Ltd., Ji’nan, Shandong, China). The antibodies to many viruses and bacteria were negative in these embryos when tested by Jinan Baizhun Biological Inspection Co., Ltd (Ji’nan, Shandong, China), including adenovirus group Ⅰ and group Ⅲ, avian influenza type A, reovirus, anemia virus, fowl pox, infectious bronchitis virus, infectious bursal disease, infectious laryngotracheitis virus, lymphoid leucosis virus A, B, and J, Marek’s disease (serotype 1, 2, and 3), *Mycoplasma gallisepticum*, *Mycoplasma synoviae*, Newcastle disease virus, reticuloendotheliosis virus, and *Salmonella pullorum* Gallinarium. The fertilized eggs were individually weighed and divided into 10 groups, each group consisting of four replicates with three eggs per replicate. The eggs were incubated under standard conditions (temperature: 38 °C, humidity: 60–70%). All eggs were candled and weighed at embryonic days 7 and 14 to eliminate undeveloped eggs. They were untreated or injected with 0.2 mL/egg of PBS, LPS (125 ng/egg; 0.2 mL/egg), PBS and ethanol (0.2 mL each per egg), quercetin + LPS (10, 20 or 40 nmoL + LPS 125 ng/egg), or quercetin (10, 20 or 40 nmol/egg) (Table 1). Each treatment was administered to 15 day old embryonated eggs via injection into the allantoic cavity according to the procedure described by a previous study [27]. The injection was conducted in a vertical clean bench after disinfection with 75% alcohol and 1% povidone iodine solution in 75% alcohol. The injection hole was sealed by paraffin before the eggs were returned to the incubator. All eggs were injected by the same individual to reduce experimental variation. The duration of eggs outside the incubator for weighing, examination, and injection of the treatment solution was approximately 10 min.

At embryonic day 19, the medial part of the duodena, whole part of the ceca, and livers of the embryos were collected for histological examination, and RNA extraction was performed for real-time quantitative polymerase chain reaction (qPCR). The tissues for histological examination were processed by a routine method (see below). The sample for PCR was stored in liquid nitrogen until RNA extraction.

### 2.2. Histology

Tissues were fixed in 4% paraformaldehyde, dehydrated, embedded in paraffin blocks, sectioned to 3 μm thick sections (model: RM2016, Shanghai Leica Instrumental Ltd., Shanghai, China), mounted on slides, and stained with standard hematoxylin and eosin (GP1031, Servicebio, Wuhan servicebio technology Co., Ltd, Wuhan, Hubei Province, China) following established histology procedures [28]. The slides were scanned by a Panoramic DESK (3D HISTECH Ltd., Empty Coolidge Ave, Budapest, Hungary) with the panoramic scanner software. Case viewer software (3D HISTECH Ltd., Empty Coolidge Ave, Budapest, Hungary) was used to take pictures. For periodic acid schiff (PAS) staining, paraffin sections (3 μm) were deparaffinized, and stained according to the manufacturer’s instructions. The photos were taken by a microscope (model: E100, Nikon, Tokyo, Japan) equipped with a Nikon Digital imaging system (model: Nikon DS-U3, Nikon, Tokyo, Japan). The color of nucleus of cell was light blue, and the glycogen presented as purple.

### 2.3. Frozen Oil Red O Staining

Liver tissues were fixed with fixative solution for 24 h at 4 °C. Subsequently, the tissues were placed, dehydrated, and precipitated with 15% sucrose solution and 30% sucrose solution at 4 °C. The surface water was removed slightly with filter paper. Then, the tissues were embedded using optimum cutting temperature compound (OCT, sakura, Catalog-number: 4583) and sliced after the OCT turned white and hard (frozen section machine, thermo, model: CYROSTAR NX50, Thermo Fisher Scientific, Waltham, MA, USA; slicer: Shanghai leica instrument Co., Ltd., model: leica 819). The slices (8 μm) were baked and dried at 60 °C, then fixed with fixative solution for 15 min, and finally stained with oil red O staining solution at room temperature for 8–10 min (stamped and kept out of light). The background color was removed twice with 60% isopropanol for 3 s and 5 s in turn, and rinsed twice by pure water for 10 s. The slices were counterstained by hematoxylin solution for 3–5 min, and then rinsed with pure water three times for 5 s, 10 s, and 30 s in turn. They were treated with differentiation solution (60% ethanol as solvent) for 2–8 s, rinsed with pure water for 10 s in turn, then put in Scott’s tap bluing for 1 s, rinsed with pure water for 5 s and 10 s in turn, and finally sealed with glycerin gelatin. The lipid droplets (tangerine to bright red) were observed under the microscope, and photos were taken at the same time.

### 2.4. qPCR

Total RNA was extracted from liquid nitrogen-frozen duodenum (50 mg) using the TransZol Up Plus RNA kit (Catalog-number: ER501-01, TransGen Biotech Co., Ltd, Beijing, China). Absorbance at 230, 260, and 280 nm was measured by spectrophotometry (NanoDrop2000, Thermo Fisher Scientific, Waltham, MA, USA) for the assessment of RNA purity. The extract with both OD260/280 nm (2.07 ± 0.03) and OD260/230 nm ratios (2.20 ± 0.12) was acceptable for PCR analysis. First-strand cDNA was synthesized from total RNA (800 ng) with an EasyScript^®^One-step gDNA removal and cDNA Synthesis SuperMix kit (Catalog-number: AE311-03, TransGen Biotech, Beijing, China) by a T100 thermal cycler (BIO-RAD Laboratories, Inc., California, CA, USA) according to the manufacturer’s protocol. The mRNA levels of genes were determined by real-time quantitative PCR using BioRad CFX Connect Real-Time system (Model No.: Connect^TM^ Optics Module, BIO-RAD Laboratories, Inc., California, CA, USA). A total of seven genes were selected to study. The sequence of genes was obtained from the USA National Center for Biotechnology Information web (NCBI, https://www.ncbi.nlm.nih.gov/nuccore/ (accessed from 5 June 2021 to 15 September 2021)), and the forward and reverse primers were obtained by Primer-BLAST (https://www.ncbi.nlm.nih.gov/tools/primer-blast/ (accessed from 5 June 2021 to 15 September 2021)). The primers are listed in Table 2. For real-time quantitative PCR, 2 μL of isolated template was added to the PCR reaction mixture, which contained 10 μL 2 × PerfectStart^®^Green qPCR SuperMix (Catalog-number: AQ601-02, TransGen Biotech, Beijing, China) and 0.2 μM of each primer (0.4 μL/primer). PCR reactions consisted of one cycle at 94 °C for 30 s and 43 cycles at 94 °C for 5 s, with an annealing temperature of 60 °C for 15 s and 72 °C for 10 s. Glyceraldehyde-3-phosphate dehydrogenase (GAPDH) was used as the housekeeping gene. The relative levels of target mRNA expression were calculated using the 2^−∆∆Ct^ method.

### 2.5. Immunohistochemistry Investigation

Immunohistochemical investigations were carried out using the indirect method of peroxidase with a primary antibody specific for AMPKα2 (AMPKα2, anti-AMPKα2, GB113685, Servicebio, Wuhan servicebio technology Co., Ltd, Wuhan, Hubei Province, China).

Paraffin sections were deparaffinized and rehydrated, and sections were kept in epitope retrieval solution (pH = 6.0 citric acid), then heated and boiled with moderate power by a microwave oven for 8 min, cooled for 8 min, and finally heated with low power for 7 min, then were washed in PBS (pH = 7.4) in a decoloration shaker for 3 times (5 min per time). Serial sections were incubated with 3% H_2_O_2_ in room temperature for 25 min in dark place, and then washed in PBS (pH = 7.4) in a decolorizing shaker three times (5 min each) to eliminate endogenous peroxidase activity.

They were then rinsed and blocked by 3% normal bovine serum albumin (G5001, Servicebio) in room temperature for 30 min. Sections were rinsed and incubated with primary antibodies, anti-AMPKα2 (1:1000, GB113685, Servicebio), in a moist chamber at 4 °C for 12 h. Sections were washed in PBS (pH = 7.4) in a decoloration shaker three times (5 min each) and centrifuged. Following this, the sections were rinsed and incubated for 50 min at room temperature with horseradish peroxidase (HRP)-conjugated anti-rabbit immunoglobulins (IgG) (1:200; GB23303, Servicebio). Sections were washed in PBS (pH = 7.4) in a decolorizing shaker three times (5 min each). The slides were incubated with diaminobenzidine solution (DAB, G1211, Servicebio), and we terminated the staining by running water. Then, they were counterstained with hematoxylin solution for 3 min and rinsed with running water. After rinsing in PBS, the slides were dehydrated, mounted, and examined under a microscope (E100, Nikon, Tokyo, Japan) equipped with a Nikon Digital imaging system (Nikon DS-U3, Nikon, Tokyo, Japan).

### 2.6. Statistical Analysis

All data were statistically analyzed using SPSS software (Version 16.0, SPSS Inc., Chicago, IL, USA); the results are presented as mean ± standard deviation, and were analyzed using the nonparametric multiple-comparisons *t*-test and ANOVA method. Histograms were drawn using GraphPad Prism software (Version 7.0, GraphPad Software, Inc., San Diego, CA, USA) [29]. The grayscale analysis was conducted using ImageJ software (Version 1.8.0) for immunohistochemistry investigation, hepatic lipid droplets, and hepatic glycogen. Differences at *p* < 0.01 were considered significant.

## 3. Results

### 3.1. Effects of Quercetin on Duodenal Inflammation after LPS Induction in the Chicken Embryos

**Figure 1:**
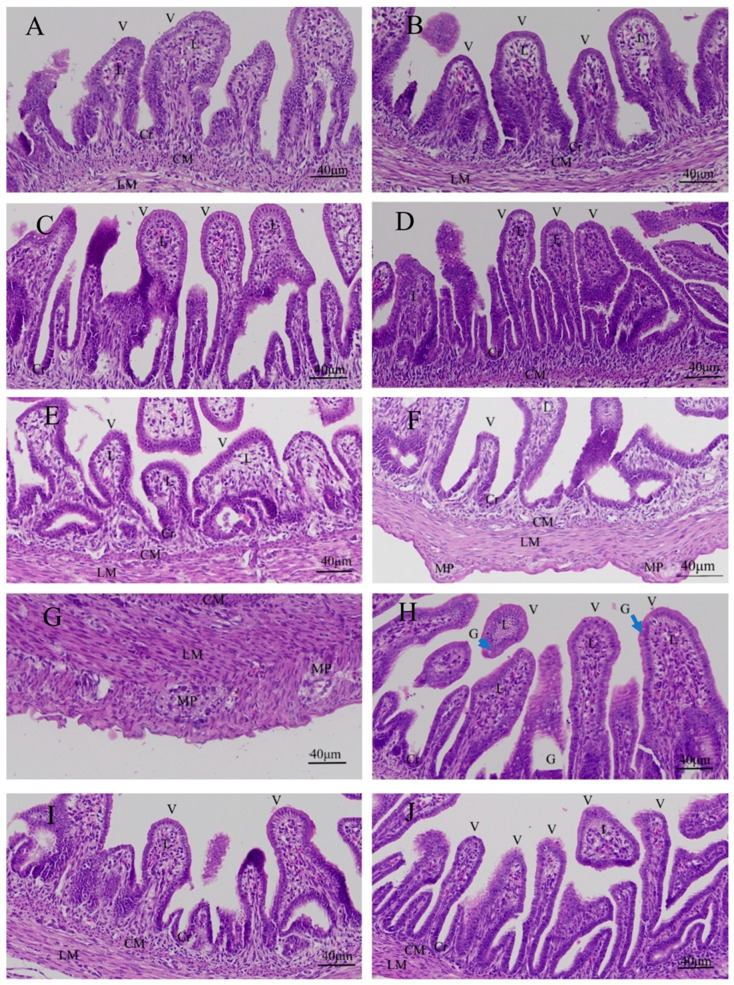
Histopathologic changes of the duodena induced by LPS and quercetin treatment in the chicken embryos: (**A**) control group; (**B**) PBS group; (**C**) PBS + ethanol group; (**D**) 10 nmol quercetin group; (**E**) 20 nmol quercetin group; (**F**) 40 nmol quercetin group; (**G**) LPS group (125 ng LPS/egg/mL); (**H**) treatment group Ⅰ (125 ng LPS/egg + 10 nmol/egg quercetin); (**I**) treatment group Ⅱ (125 ng LPS/egg + 20 nmol/egg quercetin); (**J**) treatment group Ⅲ (125 ng LPS/egg + 40 nmol/egg quercetin). There were inflammatory cell infiltrations (about 15 macrophages) in the myenteric plexus in LPS group. Scale bar (400×): 40 μm. V: villus; Cr: crypt; G: goblet cells (blue arrow); L: lamina propria; CM: muscularis externa, inner circular; LM: muscularis externa, outer longitudinal; MP: myenteric plexus. Hematoxylin and eosin staining.

There was no inflammatory response in the control group, PBS group, PBS + ethanol group, and quercetin group (Figure 1A–F). The goblet cells were found in the villi in the PBS + ethanol group and quercetin treatment group Ⅰ (Figure 1C,H). There was inflammatory cell infiltration in the MP of the LPS group (Figure 1G and Supplementary Figure S1). No inflammatory cell infiltration was presented in the LPS + Q group (Figure 1H–J).

### 3.2. Effects of Quercetin on Cecal Inflammation after LPS Induction in the Chicken Embryos

**Figure 2:**
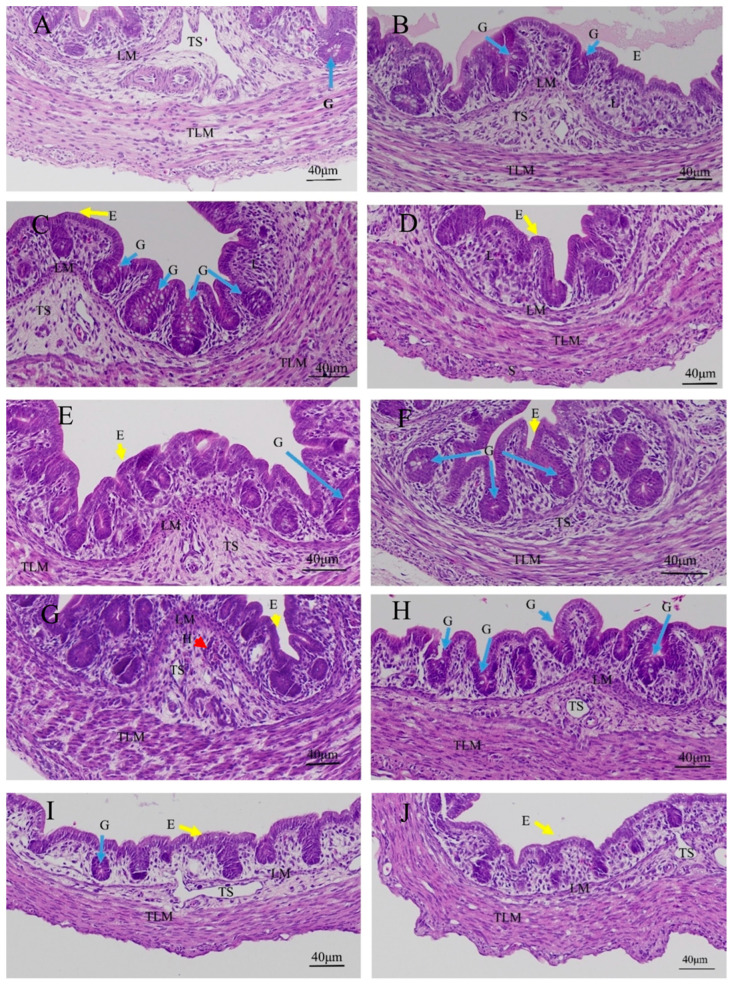
Histopathologic changes of cecum induced by LPS and treated quercetin in chicken embryos: (**A**) control group; (**B**) PBS group; (**C**) PBS + ethanol group; (**D**) 10 nmol quercetin group; (**E**) 20 nmol quercetin group; (**F**) 40 nmol quercetin group; (**G**) LPS group (125 ng LPS/egg/mL); (**H**) treatment group I (125 ng LPS/egg + 10 nmol/egg quercetin); (**I**) treatment group II (125 ng LPS/egg + 20 nmol/egg quercetin); (**J**) treatment group III (125 ng LPS/egg + 40 nmol/egg quercetin). There were inflammatory cell infiltrations (about 11 heterophils) in the submucosal layer between LM and TLM in LPS group. Scale bar (400×): 40 μm. E: epithelial cells (yellow arrow); G: goblet cells (blue arrow); L: lamina propria; LM: lamina muscularis; TLM: tunica muscularis, longitudinal layer; H: heterophils (red arrow); TS, Tela submucosa. Hematoxylin and eosin staining.

There was no inflammatory response in the control group, PBS group, PBS + ethanol group, and quercetin groups (Figure 2A–F); however, goblet cells were found in these groups (Mucin 2 is the main biomarker of a goblet cell) (Figure 2A–D,F). There were inflammatory cell infiltrations (heterophils) in the submucosal layer between LM and TLM in the LPS group; the muscle fibers of outer longitudinal muscularis were broken in the LPS group (Figure 2G and Supplementary Figure S2). No inflammatory cell infiltration was presented in the LPS + Q group (Figure 2H–J).

### 3.3. Effects of Quercetin on Hepatic Inflammation after LPS Induction in the Chicken Embryos

**Figure 3:**
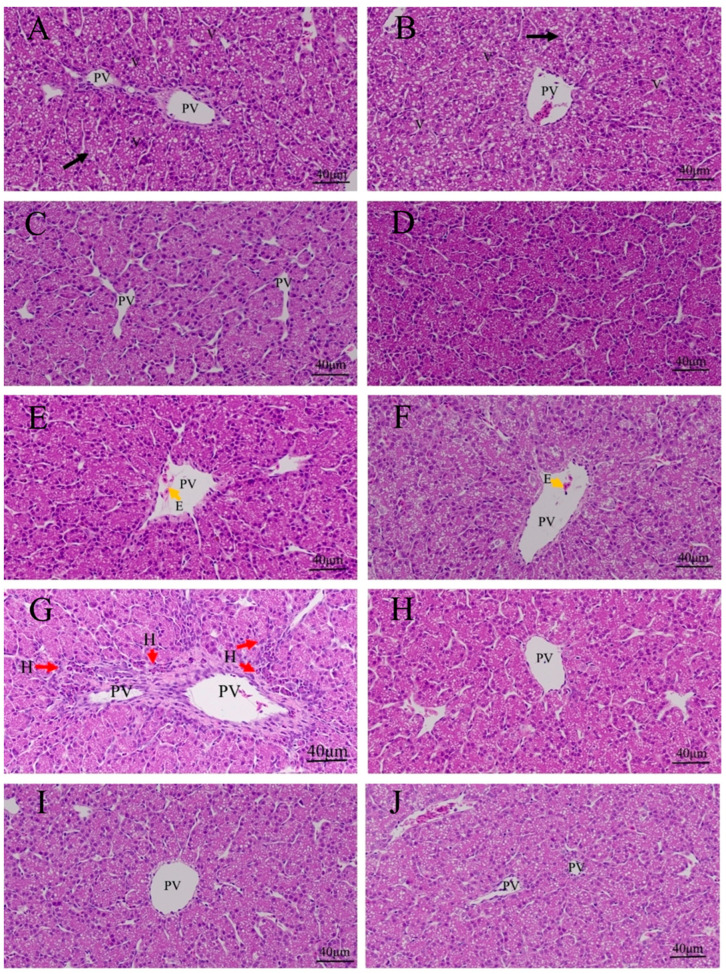
Histopathologic changes of the livers induced by LPS and treated by quercetin in the chicken embryos: (**A**) control group; (**B**) PBS group; (**C**) PBS + ethanol group; (**D**) 10 nmol quercetin group; (**E**) 20 nmol quercetin group; (**F**) 40 nmol quercetin group; (**G**) LPS group (125 ng LPS/egg/mL); (**H**) treatment group Ⅰ (125 ng LPS/egg + 10 nmol/egg quercetin); (**I**) treatment group Ⅱ (125 ng LPS/egg + 20 nmol/egg quercetin); (**J**) treatment group Ⅲ (125 ng LPS/egg + 40 nmol/egg quercetin). There were inflammatory cell infiltrations (about 21 heterophils) around portal veins in the LPS group. Scale bar (400×): 40 μm. E: erythrocyte (yellow arrow); V: vacuolization (black arrow). PV: portal vein; H, heterophils (red arrow).

There was no inflammatory response in the control group, PBS group, PBS + ethanol group, and quercetin groups (Figure 3A–F). There were inflammatory cell infiltrations around portal veins in the LPS group (Figure 3G and Supplementary Figure S3). No inflammatory cell infiltration was presented in the LPS + Q groups (Figure 3H–J).

### 3.4. Quercetin Ameliorates Inflammation through Modulating Lipid Droplet Content in the Liver of the Chicken Embyos

**Figure 4:**
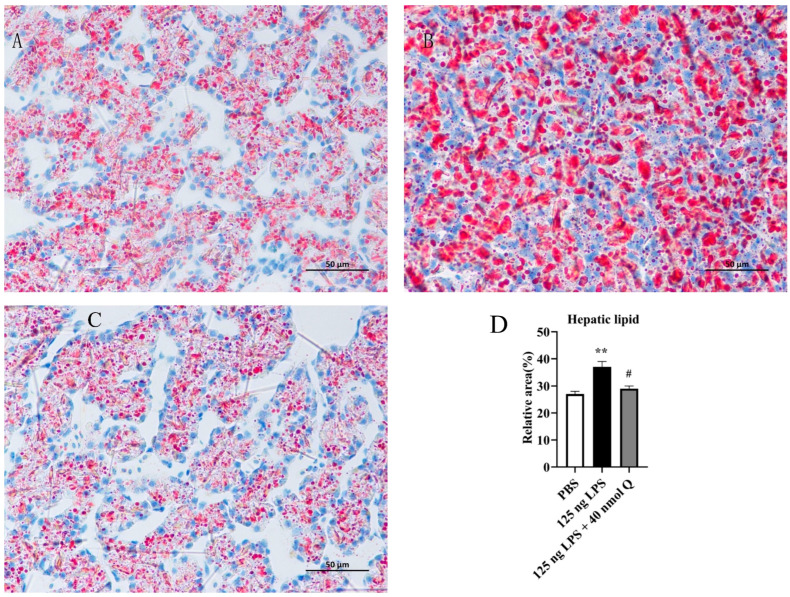
The contents of lipid droplets in liver induced by LPS (125 ng) and treated with 40 nmol/L quercetin in chicken embryos, oil red O staining (400×): (**A**) lipid droplets were present in the hepatocytes of the PBS group; (**B**) the lipid droplet content in the LPS group significantly increased when compared with the PBS group; (**C**) the lipid droplet content decreased in the treatment group (125 ng LPS + 40 nmol Q group) when compared with the LPS group; (**D**) relative lipid droplet area, i.e., the ratio of lipid droplet area to whole area in different groups. Lipid droplets were stained as tangerine to bright red. Scale bar: 50 μm. Data are expressed as the mean ± SD; significant difference between the PBS group and LPS group, ** < 0.01; significant difference between the LPS group and LPS + Q group, # < 0.05.

As expected, the lipid droplets could be found in the cytoplasm of hepatocytes in the PBS group (Figure 4A). The lipid droplet content in the LPS group significantly increased when compared with the PBS group (*p* < 0.01) (Figure 4B,D). The lipid droplet content decreased in the treatment group (125ng LPS/egg + 40 nmol Q group/egg) when compared with the LPS group (*p* < 0.05) (Figure 4C,D).

### 3.5. Quercetin Ameliorates Inflammation by Modulating Glycogen Content in the Livers of the Chicken Embryos

**Figure 5:**
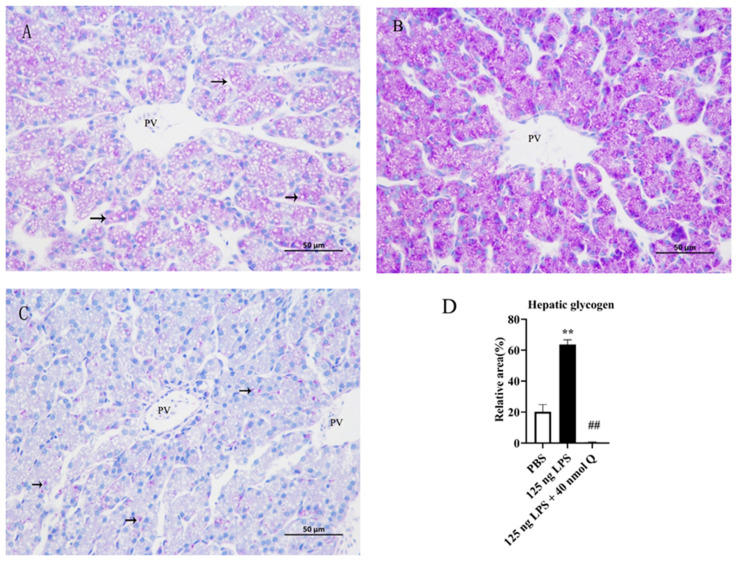
The hepatic glycogen content induced by LPS (125 ng/egg) and treated with 40 nmol/egg quercetin in chicken embryos, periodic acid Schiff staining (400×): (**A**) glycogen could be found in the hepatocytes of the PBS group; (**B**) the glycogen content in the LPS group significantly increased when compared with the PBS group; (**C**) the glycogen content significantly decreased in the treatment group (125 ng LPS/egg + 40 nmol Q/egg) when compared with the LPS group; (**D**) relative glycogen area, i.e., the ratio of glycogen area to whole area in different groups. Glycogen (black arrow: purple). Scale bar: 50 μm. PV: portal vein. Data are expressed as the mean ± SD; significant differences between the PBS group and LPS group, ** < 0.01; significant differences between the LPS group and LPS + Q group, ## < 0.01.

Glycogen could be found in the cytoplasm of hepatocytes in the PBS group (Figure 5A). The glycogen content in the LPS group significantly increased when compared with the PBS group (*p* < 0.01) (Figure 5B,D). The glycogen content significantly decreased in the quercetin treatment group when compared with the LPS group (*p* < 0.01) (Figure 5C,D).

### 3.6. Quercetin Ameliorates Inflammation through Modulating Energy Metabolism-Associated Gene mRNA Expression in the Duodena of the Chicken Embryos

**Figure 6:**
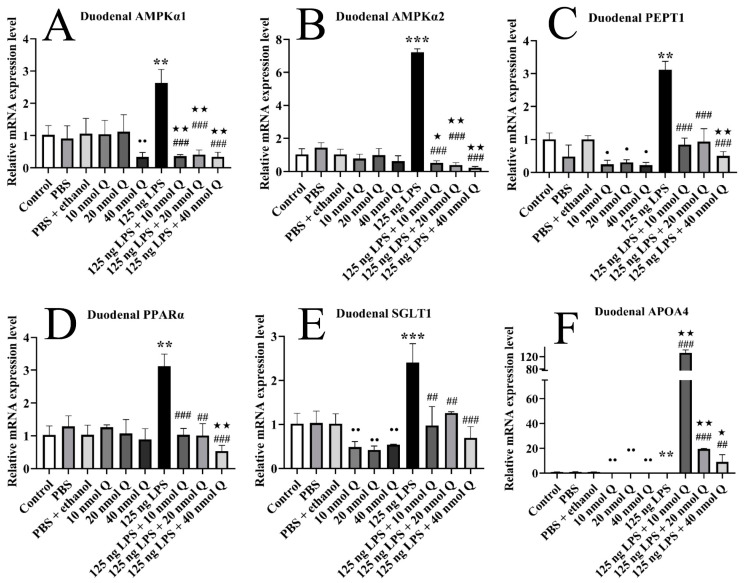
Quercetin ameliorates LPS-induced duodenal inflammation by regulating energy metabolism. Data are presented as the mean ± SD; significant difference between the PBS group and LPS group, ** < 0.01, *** < 0.001; significant difference between the LPS group and LPS + Q group, ## < 0.01, ### < 0.001; significant difference between the PBS + ethanol group and LPS + Q group, ★ < 0.05, ★★ < 0.01; significant differences between the PBS + ethanol group and Q group, • < 0.05, •• < 0.01. GAPDH was used as a housekeeping gene.

The duodenal mRNA expression of AMPKα1, AMPKα2, PEPT1, SGLT1, and PPARα was significantly upregulated 2.6-fold, 7.2-fold, 3.1-fold, 2.4-fold, and 3.1-fold when compared with the PBS groups after LPS challenge (*p* < 0.01 or *p* < 0.001), respectively, but significantly decreased upon administrating with three doses of quercetin (*p* < 0.01 or *p* < 0.001) (Figure 6A–E). The mRNA expression of APOA4 was significantly downregulated 0.1-fold after LPS induction (*p* < 0.01), but significantly upregulated by quercetin (*p* < 0.01 or *p* < 0.001) (Figure 6F).

Quercetin (10, 20, or 40 nmol) significantly decreased the duodenal mRNA expression of PEPT1, SGLT1, and APOA4 when compared with the PBS + ethanol groups (*p* < 0.01 or *p* < 0.05). Quercetin (40 nmol) significantly decreased the duodenal mRNA expression of AMPKα1 when compared with the PBS + ethanol groups (*p* < 0.01).

The mRNA expression of AMPKα1 and AMPKα2 in the LPS + quercetin groups was significantly downregulated when compared with the PBS + ethanol groups (*p* < 0.01 or *p* < 0.05), respectively. The mRNA expression of PEPT1 and PPARα in the LPS + 40 nmol quercetin group was significantly downregulated when compared with the PBS + ethanol groups (*p* < 0.01), respectively. The mRNA expression of APOA4 was significantly upregulated in the LPS + quercetin groups when compared with the PBS + ethanol groups after LPS induction (*p* < 0.01 or *p* < 0.05).

### 3.7. Quercetin Ameliorates Inflammation through Decreasing AMPKα2 Protein Expression in the Duodena of the Chicken Embryos

**Figure 7:**
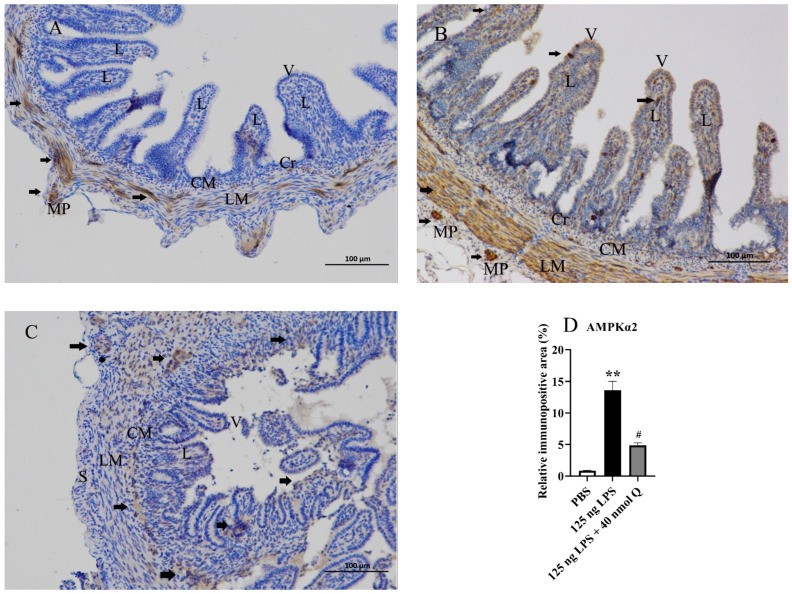
Immunohistochemical detection in HRP of AMPKα2 in the duodena induced by LPS (125 ng/egg) and treated with 40 nmoL/egg quercetin in the chicken embryos (200×): (**A**) the antibody for AMPKα2 revealed immunopositivity in the tunica muscularis and myenteric plexus of the duodena of the PBS samples, but no immunoreactivity in the intestinal villi and crypts; (**B**) immunopositivity in the villi, crypts, lamina propria, tunica muscularis, and myenteric plexus in the duodena increased after LPS induction compared with the PBS group; (**C**) the immunopositivity to AMPKα2 in the treatment group (125 ng LPS/egg + 40 nmol Q/egg) significantly decreased when compared with the PBS group; (**D**) relative AMPKα2 immunopositive area, i.e., the ratio of AMPKα2 immunopositive area to whole area in different groups. Immunopositivity to AMPKα2 (black arrow, brown and yellow). Scale bar: 100 μm. V: villus; Cr: crypt; L: lamina propria; CM: muscularis externa, inner circular; LM: muscularis externa, outer longitudinal; S: serosa; MP: myenteric plexus. Data are expressed as the mean ± SD; significant difference between the PBS group and LPS group, ** < 0.01; significant difference between the LPS group and LPS + Q group, # < 0.05.

The immunopositivity of AMPKα2 in the villi, crypts, lamina propria, tunica muscularis, and myenteric plexus in duodenum significantly increased after LPS induction when compared with the PBS group (*p* < 0.01), whereas the immunopositivity to AMPKα2 in the quercetin treatment group significantly decreased when compared with the LPS group (*p* < 0.05) (Figure 7A–D).

### 3.8. Quercetin Ameliorates Inflammation through Modulating Energy Metabolism-Associated Gene mRNA Expression in the Cecum of the Chicken Embryos

**Figure 8:**
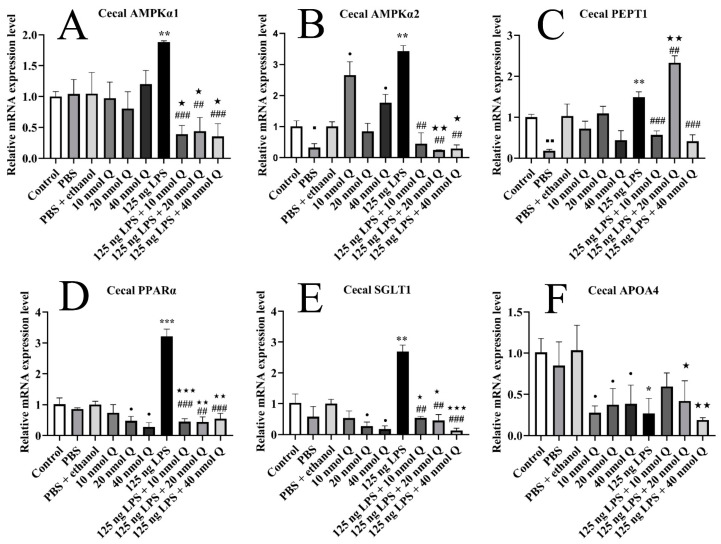
Quercetin ameliorates LPS-induced cecal inflammation by regulating energy metabolism. Data are presented as the mean ± SD; significant difference between the PBS group and control group, ■ < 0.05, ■■ <0.01; significant difference between the PBS group and LPS group, * < 0.05, ** <0.01, *** < 0.001; significant difference between the LPS group and LPS + Q group, ## < 0.01, ### < 0.001; significant difference between the PBS + ethanol group and LPS + Q group, ★ < 0.05, ★★ < 0.01, ★★★ < 0.001; significant difference between the PBS + ethanol group and Q group, • < 0.05. GAPDH was used as a housekeeping gene.

The cecal mRNA expression of AMPKα1, AMPKα2, PEPT1, SGLT1, and PPARα was significantly upregulated 1.9-fold, 3.4-fold, 1.5-fold, 2.7-fold, and 3.2-fold when compared with the PBS groups after LPS challenge (*p* < 0.01 or *p* < 0.001), respectively, but significantly decreased upon administrating three doses of quercetin (*p* < 0.01 or *p* < 0.001) (Figure 8A–E). The gene expression of APOA4 was significantly downregulated 0.3-fold after LPS induction (*p* < 0.05), but quercetin (125 ng LPS/egg + 10 nmol Q/egg or 125 ng LPS/egg + 20 nmol Q/egg) could upregulate its expression without statistical difference (Figure 8F).

Quercetin (10, 20, or 40 nmol) significantly decreased the cecal mRNA expression of APOA4 when compared with the PBS + ethanol groups (*p* < 0.05). Quercetin (20 or 40 nmol) significantly decreased the cecal mRNA expression of SGLT1 and PPARα when compared with the PBS + ethanol groups (*p* < 0.05). Quercetin (10 or 40 nmol) significantly increased the cecal mRNA expression of AMPKα2 when compared with the PBS + ethanol groups (*p* < 0.05).

The mRNA expression of AMPKα1, SGLT1, and PPARα in the LPS + quercetin groups was significantly downregulated when compared with the PBS + ethanol groups (*p* < 0.001, *p* < 0.01, or *p* < 0.05), respectively. The mRNA expression of AMPKα2 and APOA4 in the LPS + 20 nmol quercetin group or LPS + 40 nmol quercetin group was significantly downregulated when compared with the PBS + ethanol groups (*p* < 0.01), respectively. There was significant upregulation of the mRNA expression of PEPT1 in the LPS + 20 nmol quercetin groups (*p* < 0.01) when compared with the LPS group or PBS + ethanol groups.

### 3.9. Quercetin Ameliorates Inflammation through Modulating Energy Deficiency-Associated Gene mRNA Expression in the Livers of the Chicken Embryos

**Figure 9:**
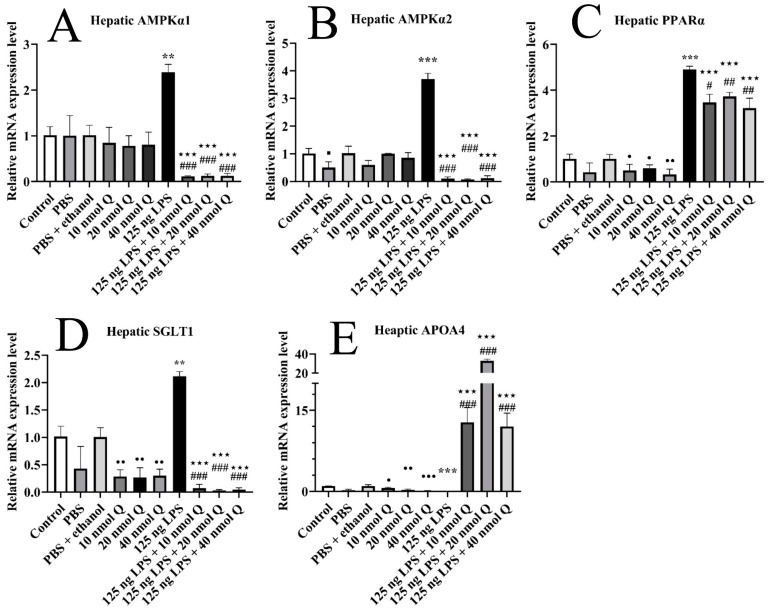
Quercetin ameliorates LPS-induced hepatic inflammation by regulating energy metabolism. Data are presented as the mean ± SD; Response: Significant difference between the PBS group and control group. ■ < 0.05;.significant difference between the PBS group and LPS group, ** < 0.01, *** < 0.001; significant difference between the LPS group and LPS + Q group, # < 0.05, ## < 0.01, ### < 0.001; significant difference between the PBS + ethanol group and LPS + Q group, ★★★ < 0.001; significant differences between the PBS + ethanol group and Q group, • < 0.05, •• < 0.01, ••• < 0.001. GAPDH was used as a housekeeping gene.

The hepatic mRNA expression of AMPKα1, AMPKα2, SGLT1, and PPARα was upregulated 2.4-fold, 3.7-fold, 2.1-fold, and 4.9-fold when compared with the PBS groups after LPS challenge (*p* < 0.01 or *p* < 0.001), respectively, but decreased upon administrating three doses of quercetin (*p* < 0.05, *p* < 0.01 or *p* < 0.001) (Figure 9A–D). The mRNA expression of APOA4 was downregulated 0.04-fold after LPS induction (*p* < 0.001), but significantly upregulated in the quercetin + LPS treatment group (*p* < 0.001).

Quercetin (10, 20, or 40 nmol) significantly decreased the hepatic mRNA expression of PPARα, SGLT1, and APOA4 when compared with the PBS + ethanol groups (*p* < 0.05, *p* < 0.01, or *p* < 0.001).

The mRNA expression of AMPKα1, AMPKα2, SGLT1, and PPARα in the LPS + quercetin groups was significantly downregulated when compared with the PBS + ethanol groups (*p* < 0.001). The mRNA expression of APOA4 in the LPS + quercetin groups was significantly upregulated when compared with the PBS + ethanol groups (*p* < 0.001).

### 3.10. Quercetin Ameliorates Inflammation through Decreasing AMPKα2 Protein Expression in the Livers of the Chicken Embryos

**Figure 10:**
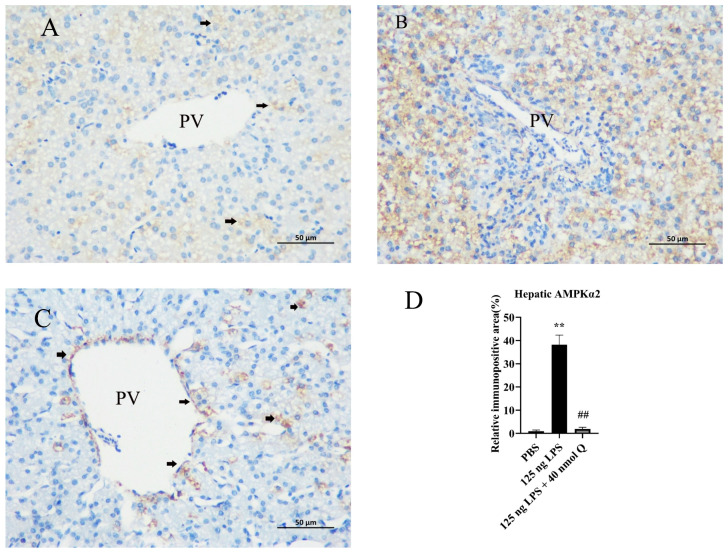
Immunohistochemical detection in HRP of AMPKα2 in the livers induced by LPS (125 ng/egg) and treated with 40 nmol/egg quercetin in the chicken embryos (400×): (**A**) sporadic immunoreactivity to AMPKα2 in the PBS group; (**B**) the immunopositivity to AMPKα2 in the cytoplasm of hepatocytes in the LPS group significantly increased when compared with the PBS group; (**C**) the immunopositivity to AMPKα2 in the treatment group (125 ng LPS/egg + 40 nmol Q/egg) significantly decreased when compared with the LPS group, presenting immunopositivity around the portal vein; (**D**) relative AMPKα2 immunopositive area, i.e., the ratio of AMPKα2 immunopositive area to whole area in different groups. Immunopositivity to AMPKα2 (black arrow, brown to yellow). Scale bar: 50 μm. PV: portal vein. Data are expressed as the mean ± SD; significant difference between the control group and LPS group, ** < 0.01; significant difference between the LPS group and LPS + Q group, ## < 0.01.

The immunopositivity to AMPKα2 in the cytoplasms of hepatocytes in the LPS group significantly increased when compared with the PBS group (*p* < 0.01), whereas the immunopositivity to AMPKα2 in the treatment group significantly decreased when compared with the LPS group (*p* < 0.01) (Figure 10A–D).

## 4. Discussion

Quercetin alleviated the inflammation in the duodena, ceca, and livers. According to the results of histopathological investigation, there was inflammatory cell infiltration (macrophages or heterophils) in the duodenum, cecum, and liver of the chicken embryos after LPS induction. Our previous study demonstrated that quercetin could balance the mRNA and protein expression of inflammatory factors in the duodena of the chicken embryos [30]. Our results found that quercetin significantly improved the inflammation of the duodena, ceca, and livers after LPS induction in the chicken embryos.

Quercetin could decrease the duodenal, cecal, and hepatic mRNA expression of energy deficient-associated genes. The present study found that quercetin (10, 20, or 40 nmol) significantly decreased the duodenal and hepatic mRNA expression of PEPT1, SGLT1, and APOA4 when compared with the control groups after LPS challenge, while quercetin (20 or 40 nmol) significantly decreased the cecal mRNA expression of PEPT1, SGLT1, and APOA4 when compared with the control groups after LPS challenge. Quercetin (40 nmol) significantly decreased the duodenal mRNA expression of AMPKα1 and cecal mRNA expression of AMPKα2 when compared with the PBS groups after LPS induction. Evidence shows that quercetin could downregulate the expression of energy deficiency-associated genes.

The limited carbohydrate content of the chicken embryos necessitates gluconeogenesis and glycogen synthesis for energy metabolism. A previous study indicated that the yolk sac is a key supplier of the nutrients; glycerol is a major substrate for synthesis of the liver and muscle glycogen in late-term chicken embryos [31]. The glycogen is depleted rapidly after LPS induction at early embryonic development, subsequently promoting gluconeogenesis and glycogen synthesis. One report indicated that the glycogen content of LPS-stimulated mouse splenocytes increased resulting from activation of glycogen synthetase I and D activity [32], which could increase glycogen production; however, there are no corresponding studies in chicken. We found that the glycogen content of the liver in the chicken embryos increased after 96 h LPS stimulation. Nevertheless, quercetin could decrease the glycogen content of the liver. The reason remains unclear. It may be associated with the inhibition of glycogen synthase kinase-3 (GSK-3) by quercetin. A previous study indicated that GSK-3 is a highly active kinase in LPS-induced sepsis [33]. Glycogen synthase kinase 3beta (GSK-3beta) is a ubiquitously expressed kinase with distinctive functions in different types of cells. One study revealed that GSK-3beta in primary human periodontal ligament cells was upregulated by LPS treatment [34]. The GSK-3beta expression may be increased in the liver after LPS induction, stimulating the glycogen synthesis increase in the liver to maintain this energetic process. This needs further research.

Our results revealed that the contents of lipid droplets in the liver increased after LPS induction in the chicken embryos. Nevertheless, quercetin decreased the lipid accumulation when compared with the LPS group. Similar results were published in rodents [35]. They found that the contents of phospholipids and triacylglycerol in rat liver increased after LPS challenge. Until now, there were no corresponding studies in chickens (*Gallus gallus*). Another study found that quercetin could ameliorate lipid deposition induced by ox-LDL in a murine macrophage cell line [36]. Therefore, the mechanism of quercetin inhibiting lipid accumulation in the liver after LPS induction remains elusive, but might be associated with the expression of PPARα; this needs further research.

Peroxisome proliferator-activated receptors (PPARs) are transcription factors that belong to the nuclear receptor superfamily. Fatty acids and many fatty acid derivatives can also directly regulate gene expression through PPARs. However, there are no reports on gene expression of PPARα after LPS challenge. PPARα is involved in lipid metabolism [37], β oxidation of the microsome, peroxysome, and mitochondria, synthesis and activation of fatty acids, glycogenesis, and bile acid metabolism. The liver presented inflammation, inhibited the proliferation of intestinal crypts, and limited the absorption of fat in PPARα-deficient mice fed high-fat diets [38], while hepatic adipose infiltration and oxidative stress were observed after 36 h fasting in PPARα-knockout mice. The fatty acid uptake and oxidation were inhibited, while ketogenesis and gluconeogenesis were impaired in PPARα-knockout mice. Liver is the primary organ involved in energy metabolism because it can metabolize fatty acid and glucose. PPARα is predominantly expressed in the liver, where it regulates energy metabolism via fatty acid oxidation [39]. Our results showed that PPARα mRNA expression was upregulated after LPS challenge in the duodena, ceca, and livers of the chicken embryos, and the lipid droplets of livers increased after LPS induction, confirming the correlation of PPARα gene expression with lipid metabolism. One study indicated that a PPARα agonist induced hepatomegaly in mice, resulting in elevated serum cholesterol, phospholipids, and triglycerides when compared with PPARα-null mice [40]. PPARα is highly expressed in the small intestine, where it serves as a master regulator of fatty acid catabolism, regulates intestinal cholesterol efflux and motility, and regulates various transporters (SGLT1 and GLUT2) and enzymes involved in fatty acid uptake and oxidation [41]. In the present study, the energy deficiency of the duodena, ceca, and livers was induced by the LPS, stimulating an increase in PPARα and SGLT1 mRNA expression, while quercetin significantly decreased this mRNA expression.

AMPK, a major factor regulating energy metabolism and autophagy, is a conserved sensing sentinel that maintains energy balance, cell growth, and protein synthesis. When energy deficiency, nutrient deprivation, and inflammation occur, the energy-sensitive AMPKα1 and AMPKα2 genes can be activated [42]. A previous study indicated that the level of ATP, ADP, and AMP in myocardiocytes decreased after LPS induction [43]. In the present study, the energy-deficient state of the duodenum, cecum, and liver was induced by the LPS, stimulating an increase in AMPKα1 and AMPKα2 mRNA and protein expression, whereas quercetin significantly decreased this expression. Thus, LPS induced energy deficiency in multiple organs, activating the mRNA and protein expression of AMPKα1 and AMPKα2. Meanwhile, mitochondrial oxidative stress accelerated fatty acid oxidation and activated the mRNA expression of PPARα after LPS challenge, inducing increases in hepatic glycogen and lipid droplets. This suggests that the energy deficiency stimulated the AMPKα1 and AMPKα2 increase after LPS challenge, and triggered glycogen synthesis and lipid accumulation. Quercetin could improve energy deficiency, decrease the mRNA and protein expression of AMPKA2, and decrease the lipid peroxidation of the duodena and liver in the chicken embryos.

PEPT1 is a vital member of the proton-dependent oligopeptide transporter family, distributed on the apical surface of many cells, such as the brush border and basolateral membrane of enterocytes [44,45]. It is crucial in the uptake of dipeptides and tripeptides. Hu and colleagues found that PEPT1-knockout mice lacked the expression of PEPT1 protein in the intestine, with reduced intestinal uptake of dipeptides [46]. Another study indicated that PEPT1-deficient mice fed a high-fat diet had a reduced caloric intake, and increased energy content in feces [47]. The PEPT1 expression was modulated by physical conditions, such as fasting, feeding, and embryo development. Ma reported that the protein expression of PEPT1 in the small intestine increased about twofold in the fasted mice [48]. The PEPT1 mRNA was expressed from embryonic day 15 to day 20 in the duodena and ceca of chicken embryos [49]. However, there are no corresponding studies on the gene expression of PEPT1 after LPS induction in chicken. In the present study, the mRNA expression of PEPT1 in the duodena and ceca was upregulated after LPS induction on embryonic day 19, whereas quercetin could decrease the duodenal mRNA expression of PEPT1 with three doses.

SGLT1, the main sodium and glucose cotransporter widely distributed in animals, has high expression in small intestine [50]. SGLT1 is located in the brush border and the basolateral membranes of enterocytes involved in sodium ion, glucose, and water absorption at different glucose concentrations [51]. Mutation or deletion of SGLT1 results in diarrhea associated with malabsorption of glucose and electrolytes. Because chickens are precocious animals, their gastrointestinal tracts are suitable for absorbing nutrients during late embryonic development and after hatch. The mRNA of SGLT1 was expressed in the villi and crypts of enterocytes from embryonic day 15 to hatch in broilers [52], consistent with our results. On embryonic day 15, intestinal villi begin to secrete maltase, aminopeptidase, SGLT1, and ATPase. The activity of these enzymes is low from embryonic days 15 to 17, and then the embryo begins to ingest amniotic fluid, which contains growth hormones and glucocorticoids that may stimulate the development of villi. On embryonic day 19, intestinal villi continue to develop, and the activities of maltase, aminopeptidase, SGLT1, and ATPase increase [53]. A previous study indicated that LPS increased SGLT1 activity in Caco-2 cell culture with a high dose of glucose [54]. We found that the mRNA expression of SGLT1 in the duodena and ceca increased after LPS challenge, while quercetin could decrease the expression.

APOA4 is one of the genes regulating fat deposition in animals, which is mainly involved in the regulation of the dynamic balance of lipid, triglyceride catabolism, glycogenesis inhibition of liver cells, glucose absorption promotion of adipocytes, enhanced insulin secretion, and resistance to inflammation and atherosclerosis [55]. It is associated with cytosolic lipid droplets, and it plays a critical role in lipidation in very low-density lipoprotein and chylomicron assembly. Overexpression of APOA4 in an intestine cell increased the size of secreted chylomicrons [56]. It was reported that APOA4 was the top differentially expressed gene at 2 weeks and 6 weeks postpartum in cow, needed to mobilize lipid storage and lipolysis during lactation [57]. APOA4-knockout rats exhibited enhanced glycolysis, attenuated gluconeogenesis, and elevated de novo lipogenesis [58]. Our results show that the mRNA expression of APOA4 was downregulated in the duodena, ceca, and livers of the chicken embryos after LPS induction. This may be associated with triglyceride metabolism and APOA4 gene methylation level in the chicken embryos during incubation. From chicken embryonic days 0 to 13, the activity of diacylglycerol transferase was enhanced, synthesizing diacylglycerol to triglyceride, and the total triglyceride concentration increased, whereas it decreased from embryonic day 18. The lipid in the yolk sac of the chicken embryo is mainly bound to very-low-density lipoprotein, which is absorbed and utilized by the yolk membrane and endodermal epithelial cells through clathrin-mediated endocytosis. This suggests that the nutrition supply of the chicken embryos depends mainly on the yolk sac during incubation, and that the level of APOA4 decreases with reduction in triglyceride level. However, nutritional requirements increased after LPS induction, but triglyceride levels in the chicken embryos decreased at embryonic day 19, triggering APOA4 mRNA expression. Another reason is associated with DNA methylation of the APOA4 gene. One study found that the APOA4 gene of human small intestine, liver tissue, and leukocytes had high methylation levels (80%, 70%, and 88%, respectively). Methylation levels of APOA4 promoters in intestinal epithelial cells can reach 60%, which leads to low APOA4 mRNA expression in these tissues and cells. Therefore, the downregulation of APOA4 mRNA expression in the duodena, ceca, and livers of chicken embryos might be associated with triglyceride metabolism or DNA methylation level. We found that quercetin could upregulate the mRNA expression of APOA4 in the duodena, ceca, and livers in chicken embryos, consistent with the results of a previous study [59].

## 5. Conclusions

We found that inflammatory cell infiltration after LPS induction in the duodena, ceca, and livers of the chicken embryos, along with induced energy deficiency, promoted the increase in hepatic lipid droplets and glycogen content in the chicken embryos. However, quercetin alleviates the visceral inflammation response, while it ameliorates inflammation through modulating energy metabolism-associated gene mRNA expression in the duodena, ceca, and livers of the chicken embryos. In addition, quercetin decreased the protein expressions of duodenal and hepatic AMPKα2 after LPS induction. In conclusion, quercetin alleviates visceral inflammation after LPS induction by improving the state of energy deficiency. The LPS-induced inflammation model was established to avoid the effect of LPS exposure from the environment and intestinal flora. The results form the basis of quercetin pretreatment (in ovo infection) to improve the energy state of chicken embryos and improve the inflammation response.

## Figures and Tables

**Table 1:** The groups of experimental animals.

Groups	The Number of Samples	Treatment	Dosage
Control group	12	No	No
PBS group	12	PBS	0.2 mL PBS/chicken embryo
PBS + ethanol group	12	PBS + ethanol	(0.2 mL PBS + 0.2 mL ethanol)/chicken embryo
125 ng LPS group	12	0.625 μg LPS/mL	0.2 mL/chicken embryo, 125 ng LPS/chicken embryo
10 nmoL Q group	12	50 μM quercetin	0.2 mL/chicken embryo, 10 nmoL Q/chicken embryo
20 nmoL Q group	12	100 μM quercetin	0.2 mL/chicken embryo, 20 nmoL Q/chicken embryo
40 nmoL Q group	12	200 μM quercetin	0.2 mL/chicken embryo, 40 nmoL Q/chicken embryo
125 ng LPS + 10 nmoL Q group	12	LPS + 10 nmol quercetin	(0.2 mL 125 ng LPS + 0.2 mL 10 nmoL Q)/chicken embryo
125 ng LPS + 20 nmoL Q group	12	LPS + 20 nmol quercetin	(0.2 mL 125 ng LPS + 0.2 mL 20 nmoL Q)/chicken embryo
125 ng LPS + 40 nmoL Q group	12	LPS + 40 nmol quercetin	(0.2 mL 125 ng LPS + 0.2 mL 40 nmoL Q)/chicken embryo

**Table 2:** Primers used in real-time quantitative polymerase chain reaction.

Genes Name	Primer Sequence (5′–3′)	Gene Bank ID	Amplicon Size (bp)
AMPKα1	F: GTGGCATTTGGGGATACGGA; R: GTTGCAGTCCCAGACTTCGT	NM_001039603.2	252
AMPKα2	F: GTGCACCGAGTCAGAAGTGA; R: CGTCCATGAAGGAGCCAGTT	NM_001039605.2	158
APOA4	F: AGCACTCAGGATGTCGCCTA; R: GTTGGTCCACGGTCTCCTTG	NM_204938.3	152
GAPDH	F: GCTAAGGCTGTGGGGAAAGT; R: TCAGCAGCAGCCTTCACTAC	NM_204305.2	161
PEPT1	F: GCAGGGATCGAGATGGACAC; R: CAAAAGAGCAGCAGCAACGA	KF366603.1	238
PPARα	F: GCAAGATGCTGCGTGAAGTG; R: TCCTCCAGGGGAGTAAGTGG	NM_001001464.1	156
SGLT1	F: TGTGGGCATAGCAGGAACAG; R: GCTTCCTCAGATACTCCGGC	NM_001293240.2	152

Abbreviation: AMPKα1: protein kinase AMP-activated catalytic subunit alpha 1; AMPKα2: protein kinase AMP-activated catalytic subunit alpha 2; APOA4: apolipoprotein A4; GAPDH: glyceraldehyde-3-phosphate dehydrogenase; PEPT1: peptide transporter 1; PPARα: peroxisome proliferator-activated receptor alpha; SGLT1: sodium–glucose cotransporter 1.

## Data Availability

Not applicable.

## Supplementary Materials

The following supporting information can be downloaded at: https://www.mdpi.com/article/10.3390/ani13132051/s1, Figure S1: The Histopathologic changes of the duodena induced by LPS and treated quercetin in chicken embryos (800×): Macrophage(black arrow); LM: muscularis externa, outer longitudinal; MP: myenteric plexus. Hematoxylin and eosin staining. Figure S2: The Histopathologic changes of the ceca induced by LPS and treated quercetin in chicken embryos (800×): H, heterophils. Figure S3: The Histopathologic changes of the livers induced by LPS and treated quercetin in chicken embryos (800×): H, heterophils (red arrow); PV: portal vein.
