# Supplementary material for: Quercetin Alleviates Inflammation and Energy Deficiency Induced by Lipopolysaccharide in Chicken Embryos
Source: Animals (Basel). 2023 Jun 21;13(13):2051. doi: 10.3390/ani13132051 (PMC10339971; doi:10.3390/ani13132051)
Supplement: Supplementary file 1 [file animals-13-02051-s001.zip › animals-2357529-supplementary.pdf]

# The legends of supplementary Figure

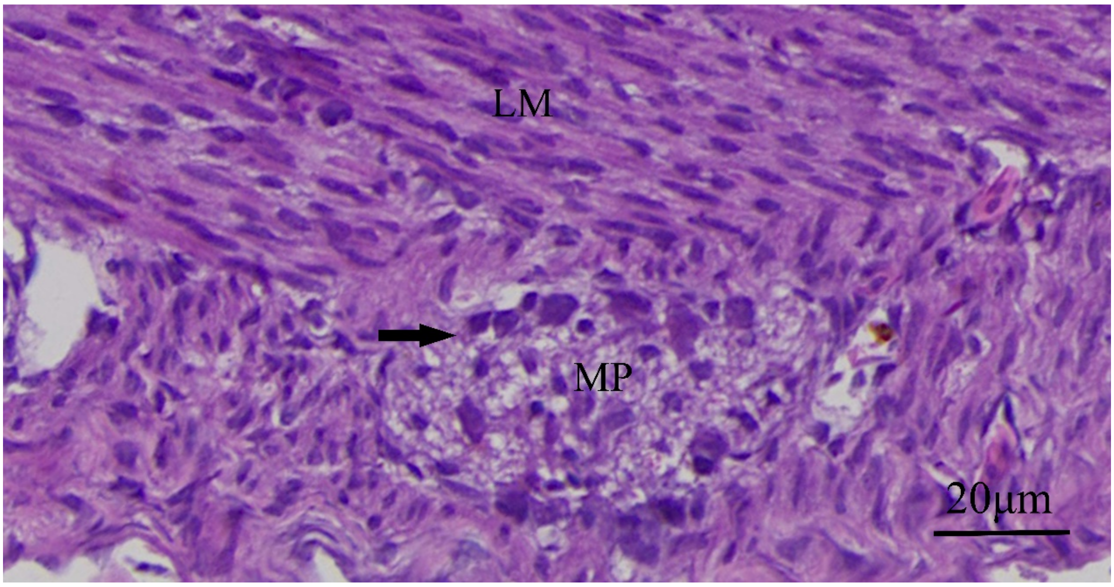


Supplementary Figure S1: The Histopathologic changes of the duodena induced by LPS and treated quercetin in chicken embryos (800×)

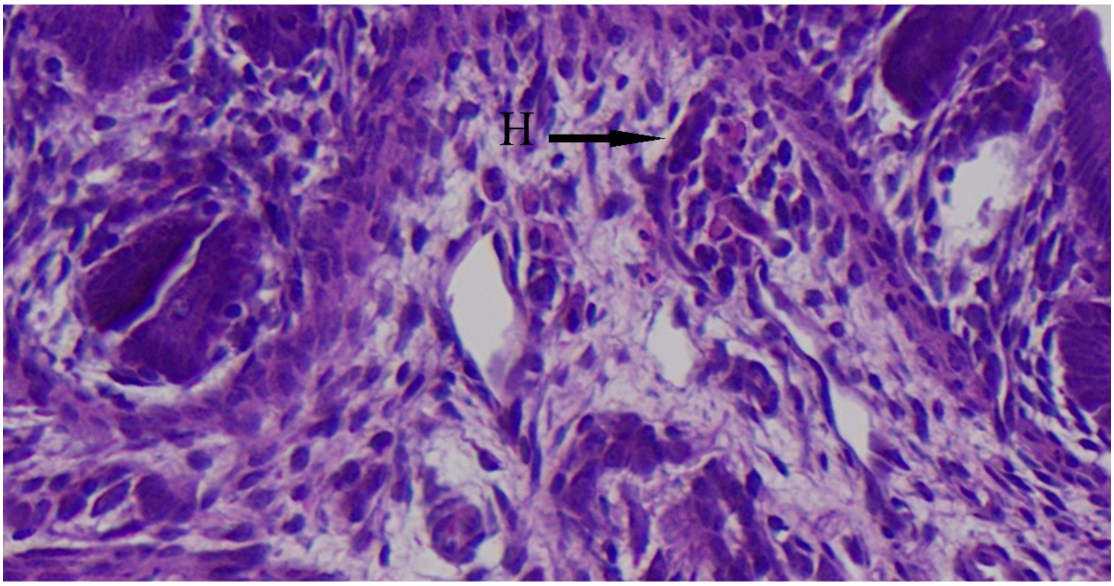


Supplementary Figure S2: The Histopathologic changes of the ceca induced by LPS and treated quercetin in chicken embryos (800×)

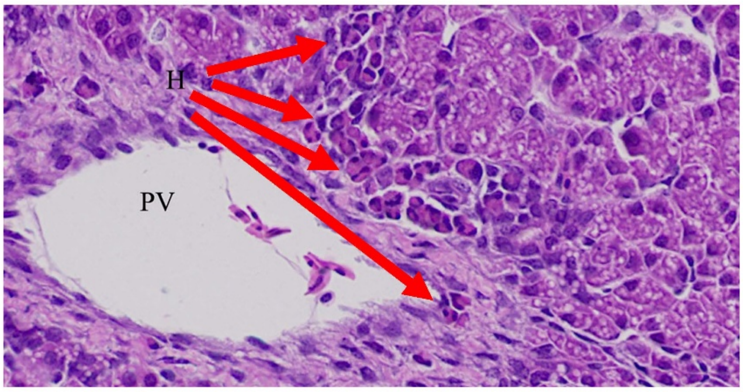


Supplementary Figure S3: The Histopathologic changes of the livers induced by LPS and treated quercetin in chicken embryos (800×)
